# Supplementary material for: Actionable tests and treatments for patients with gastrointestinal cancers and historically short median survival times
Source: PLoS One. 2022 Nov 2;17(11):e0276492. doi: 10.1371/journal.pone.0276492 (PMC9629612; doi:10.1371/journal.pone.0276492)
Supplement: S1 Data — (PDF) [file pone.0276492.s001.pdf]

Pt #	Diagnosis	Gender	Age	Status	Prior_Tx	WBC	LY#	ANC	MON#
1	Cholangio	M	74	Deceased	No	11.8	1.9	8.9	1.0
2	Cholangio	F	66	Deceased	No	5.9	1.5	3.6	0.8
3	Cholangio	F	71	Deceased	No	4.2	1.3	2.2	0.7
4	Cholangio	M	87	Deceased	No	13.6	1.5	11.1	1.0
5	Cholangio	M	72	Censored	No	12.2	2.1	9.1	1.0
6	Cholangio	M	74	Censored	No	8.6	1.0	6.5	1.1
7	Cholangio	M	67	Still Living	No	8.2	1.8	5.3	0.9
8	Cholangio	M	54	Still Living	No	9.4	1.4	7.4	0.5
9	Cholangio	F	65	Deceased	No	14.1	1.3	11.5	1.3
10	Cholangio	F	76	Censored	No	7.3	2.0	5.1	0.2
11	Cholangio	M	52	Deceased	No	18.2	2.3	15.0	0.9
12	Cholangio	F	60	Deceased	No	7.3	3.1	4.5	0.3
13	Cholangio	F	84	Deceased	No	9.3	2.2	6.8	0.3
14	Cholangio	M	64	Censored	No	4.5	2.2	2.1	0.4
15	Cholangio	M	82	Censored	No	12.3	1.3	10.8	0.2
16	Cholangio	M	71	Censored	No	7.0	2.2	4.2	0.6
17	Cholangio	F	64	Deceased	Yes	5.5	1.0	4.2	0.3
18	Cholangio	F	54	Censored	Yes	4.3	1.0	3.0	0.3
19	Cholangio	F	46	Deceased	Yes	6.7	2.3	3.7	0.6
20	Cholangio	M	68	Censored	Yes	6.3	1.7	3.9	0.7
21	Cholangio	M	71	Censored	Yes	6.6	1.0	5.1	0.4
22	Cholangio	M	69	Deceased	Yes	11.4	1.5	9.2	0.7
23	Cholangio	M	60	Censored	Yes	7.3	1.0	5.7	0.8
24	Cholangio	F	70	Censored	Yes	7.3	1.3	5.5	0.5
25	Cholangio	M	66	Still Living	Yes	19.1	1.5	17.1	0.5
26	Cholangio	F	65	Censored	Yes	11.5	2.1	8.0	0.7
27	Cholangio	F	70	Censored	Yes	4.0	0.7	2.9	0.4
28	Cholangio	F	83	Deceased	Yes	5.9	1.7	4.0	0.2
29	Cholangio	F	46	Deceased	Yes	5.0	0.7	4.1	0.2
30	Cholangio	F	86	Deceased	Yes	6.8	1.8	4.4	0.6
31	Cholangio	M	57	Deceased	Yes	11.5	0.9	10.0	0.6
32	Cholangio	M	43	Still Living	Yes	5.8	2.2	4.0	0.3
33	Cholangio	M	66	Still Living	Yes	5.9	1.6	3.9	0.4
34	Cholangio	F	57	Censored	Yes	10.6	1.1	9.1	0.5
35	Cholangio	F	45	Still Living	Yes	4.3	1.6	2.3	0.4
36	Colon	M	59	Deceased	No	8.7	1.4	6.8	0.4
37	Colon	M	70	Deceased	No	7.9	1.6	5.7	0.7
38	Colon	F	72	Deceased	No	5.7	1.9	3.4	0.4
39	Colon	F	70	Deceased	No	14.6	3.2	11.2	0.2
40	Colon	F	44	Censored	No	6.0	1.2	4.6	0.3
41	Colon	F	70	Deceased	No	9.6	2.0	7.3	0.3
42	Colon	F	42	Deceased	No	10.3	2.6	7	0.7
43	Colon	F	54	Still Living	No	10.0	2.9	6.2	1
44	Colon	F	38	Deceased	No	8.7	1.7	6.6	0.4
45	Colon	M	72	Censored	No	8.7	2.0	6.4	0.3

46	Colon	M	61	Still Living	No	10.0	1.6	7.9	0.4
47	Colon	M	52	Deceased	No	8.0	1.1	6.4	0.5
48	Colon	F	80	Still Living	No	7.4	2.2	4.9	0.4
49	Colon	M	59	Censored	No	9.2	1.6	7.4	0.7
50	Colon	M	65	Deceased	Yes	12.6	1.7	10.4	0.5
51	Colon	M	40	Censored	Yes	5.5	2.2	3.1	0.1
52	Colon	M	66	Deceased	Yes	7.7	1.3	6.1	0.3
53	Colon	M	44	Deceased	Yes	5.5	2.3	3	0.3
54	Colon	F	43	Deceased	Yes	5.2	1.2	3.6	0.4
55	Colon	M	39	Deceased	Yes	5.4	1.7	3.1	0.6
56	Colon	M	59	Censored	Yes	6.3	2.1	3.5	0.3
57	Colon	M	46	Censored	Yes	8.0	1.3	6.5	0.2
58	Colon	F	64	Deceased	Yes	5.6	0.6	4.7	0.3
59	Colon	M	54	Still Living	Yes	7.5	1.0	6.2	0.5
60	Colon	F	59	Deceased	Yes	9.6	1.3	8.1	0.4
61	Colon	F	59	Deceased	Yes	10.6	3.8	6.1	0.7
62	Colon	M	49	Deceased	Yes	6.2	1.5	4.1	0.6
63	Colon	F	53	Deceased	Yes	7.0	0.7	5.5	0.8
64	Colon	M	41	Censored	Yes	5.7	0.8	4.3	0.6
65	Colon	F	38	Deceased	Yes	11.6	3.5	7.2	0.9
66	Colon	F	55	Deceased	Yes	8.9	1.4	7	0.5
67	Colon	M	66	Censored	Yes	6.4	1.6	4.3	0.5
68	Colon	M	47	Deceased	Yes	5.1	1.1	3.5	0.5
69	Colon	M	57	Deceased	Yes	7.4	1.2	6.1	0.1
70	Colon	M	40	Deceased	Yes	11.3	2.1	8.9	0.3
71	Colon	M	62	Deceased	Yes	3.2	0.6	2.4	0.2
72	Colon	M	60	Deceased	Yes	9.3	1.6	6.9	0.8
73	Colon	F	67	Still Living	Yes	22.0	2.9	18.5	0.6
74	Colon	M	59	Deceased	Yes	7.1	1.3	5.4	0.4
75	Colon	M	51	Still Living	Yes	6.8	1.6	4.4	0.8
76	Colon	F	81	Still Living	Yes	3.8	1.0	2.5	0.3
77	Colon	M	75	Deceased	Yes	18.8	2.6	16	0.2
78	Colon	M	53	Deceased	Yes	3.9	0.9	2.9	0.3
79	Colon	F	65	Deceased	Yes	4.3	1.0	3	0.3
80	Colon	M	51	Deceased	Yes	6.7	1.7	4.4	0.6
81	Colon	M	53	Censored	Yes	4.5	1.3	3	0.2
82	Colon	F	62	Deceased	Yes	3.1	1.6	1	0.5
83	Colon	F	71	Censored	Yes	16.0	2.4	12.2	1.4
84	Colon	F	53	Deceased	Yes	7.4	1.0	6	0.4
85	Colon	F	61	Censored	Yes	9.0	1.8	6.7	0.5
86	Colon	M	59	Deceased	Yes	4.0	0.9	2.8	0.3
87	Colon	F	79	Deceased	Yes	6.5	1.2	4.8	0.5
88	Colon	M	63	Deceased	Yes	8.0	1.7	5.7	0.6

89	Colon	M	64	Censored	Yes	5.5	1.7	3.5	0.3
90	Colon	F	56	Censored	Yes	7.5	1.4	5.4	0.7
91	Colon	F	71	Deceased	Yes	8.1	1.7	6.2	0.3
92	Colon	M	56	Deceased	Yes	6.0	1.3	4	0.6
93	Colon	F	53	Still Living	Yes	8.9	2.2	6.6	0.2
94	Colon	F	54	Deceased	Yes	4.5	0.9	3.3	0.4
95	Colon	F	58	Censored	Yes	6.9	1.5	5.1	0.3
96	Colon	F	70	Deceased	Yes	8.6	2.7	5.2	0.7
97	Colon	M	56	Deceased	Yes	9.0	2.2	6.7	0.2
98	Colon	F	67	Deceased	Yes	5.1	1.6	3.1	0.4
99	Colon	F	54	Deceased	Yes	6.8	1.8	4.3	0.7
100	Ovarian		66	Still Living	No	7.6	2.5	4.5	0.7
101	Ovarian		88	Censored	No	7.0	1.7	4.7	0.5
102	Ovarian		75	Deceased	No	13.6	1.4	33.1	0.1
103	Ovarian		47	Censored	No	6.8	1	5.6	0.2
104	Ovarian		60	Deceased	No	3.0	1.0	3.1	0.1
105	Ovarian		67	Deceased	No	4.6	1.4	2.8	0.4
106	Ovarian		74	Deceased	No	4.7	0.5	4.1	0.1
107	Ovarian		60	Censored	No	9.8	2.1	7.6	0.4
108	Ovarian		55	Deceased	No	5.7	1.6	3.8	0.3
109	Ovarian		55	Censored	No	7.2	1.9	4.8	0.3
110	Ovarian		59	Deceased	No	16.3	1.7	13.7	0.9
111	Ovarian		72	Censored	No	8.1	2.7	4.3	1.1
112	Ovarian		82	Still Living	No	2.7	1.6	1.7	0.2
113	Ovarian		46	Censored	Yes	3.7	1.4	1.9	0.3
114	Ovarian		41	Deceased	Yes	3.1	0.8	2.1	0.2
115	Ovarian		65	Still Living	Yes	8.2	2.0	5.4	0.8
116	Ovarian		53	Still Living	Yes	6.5	1.8	4.4	0.5
117	Ovarian		50	Deceased	Yes	8.3	1.5	5.9	1
118	Ovarian		77	Deceased	Yes	5.8	1.6	3.6	0.5
119	Ovarian		68	Deceased	Yes	9.5	2.1	6.7	0.7
120	Ovarian		73	Deceased	Yes	8.3	2.2	6.0	0.1
121	Ovarian		67	Deceased	Yes	6.3	1.6	4.4	0.3
122	Ovarian		72	Censored	Yes	8.4	1.9	5.9	0.6
123	Ovarian		59	Deceased	Yes	3.4	0.9	2.1	0.3
124	Ovarian		61	Censored	Yes	7.2	0.9	5.8	0.4
125	Ovarian		59	Deceased	Yes	19.4	1.3	17.7	0.4
126	Ovarian		70	Still Living	Yes	14.1	1.9	3.2	0.6
127	Ovarian		74	Deceased	Yes	9.1	3.0	5.7	0.4
128	Ovarian		59	Deceased	Yes	11.8	1.2	10.3	0.3
129	Ovarian		63	Deceased	Yes	7.9	1.6	6.1	0.2
130	Ovarian		73	Deceased	Yes	5.5	1.0	4.0	0.4
131	Ovarian		75	Deceased	Yes	23.3	1.4	21.1	0.8
132	Ovarian		71	Still Living	Yes	2.9	0.6	2.1	0.3
133	Ovarian		45	Deceased	Yes	2.5	0.5	1.8	0.4
134	Ovarian		74	Deceased	Yes	7.5	2.2	5.3	0.7

135	Ovarian		55	Deceased	Yes	6.4	1.6	4.6	0.4
136	Ovarian		47	Deceased	Yes	7.6	1.5	5.8	0.3
137	Ovarian		51	Deceased	Yes	4.5	1.4	2.7	0.4
138	Ovarian		69	Censored	Yes	5.6	1.8	3.5	0.3
139	Ovarian		65	Deceased	Yes	1.7	0.8	0.7	0.2
140	Ovarian		66	Deceased	Yes	4.6	1.3	3.2	0.4
141	Ovarian		63	Censored	Yes	3.0	2.5	1.9	0.6
142	Ovarian		47	Deceased	Yes	11.5	1.4	8.9	1.1
143	Ovarian		55	Deceased	Yes	6.2	2.6	3.1	0.5
144	Ovarian		51	Deceased	Yes	7.7	1.3	6.2	0.2
145	Ovarian		39	Censored	Yes	7.0	1.0	5.5	0.4
146	Ovarian		72	Censored	Yes	6.4	1.1	5.0	0.3
147	Ovarian		62	Deceased	Yes	7.1	2.1	4.4	0.6
148	Ovarian		62	Deceased	Yes	8.2	1.9	5.6	0.7
149	Ovarian		45	Deceased	Yes	10.0	1.3	8.1	0.6
150	Ovarian		66	Censored	Yes	10.1	2.6	7.3	0.3
151	Ovarian		59	Censored	Yes	5.0	1.1	3.7	0.3
152	Ovarian		47	Still Living	Yes	10.1	2.2	7.2	0.7
153	Ovarian		51	Deceased	Yes	14.8	3.8	9.2	1.9
154	Ovarian		66	Deceased	Yes	6.8	1.5	5.0	0.3
155	Ovarian		56	Deceased	Yes	4.7	0.8	3.5	0.4
156	Ovarian		71	Still Living	Yes	10.6	2.3	8	0.2
157	Ovarian		48	Deceased	Yes	4.9	1.5	3.0	0.4
158	Ovarian		73	Deceased	Yes	11.2	1.5	9.7	0.5
159	Ovarian		70	Deceased	Yes	2.5	1	1.5	0.1
160	Ovarian		59	Deceased	Yes	3	0.9	2	0.1
161	Ovarian		68	Deceased	Yes	12.0	1.9	9.1	1.1
162	Ovarian		74	Censored	Yes	8.0	2.1	5.2	0.7
163	Ovarian		69	Censored	Yes	2.4	0.6	1.6	0.1
164	Ovarian		76	Deceased	Yes	9.5	2.7	6.3	0.4
165	Ovarian		52	Deceased	Yes	4.8	1.4	3.1	0.4
166	Ovarian		64	Deceased	Yes	9.3	3.2	5.4	0.7
167	Ovarian		48	Deceased	Yes	7.4	1.5	5.6	0.3
168	Ovarian		58	Deceased	Yes	4.0	1.1	2.7	0.3
169	Ovarian		66	Deceased	Yes	2.8	1.6	0.9	0.4
170	Ovarian		68	Deceased	Yes	5.0	1.2	3.7	0.2
171	Ovarian		76	Still Living	Yes	15.8	2	12.9	0.9
172	Ovarian		54	Deceased	Yes	4.0	0.7	3.0	0.3
173	Ovarian		66	Deceased	Yes	12.1	1.7	9.5	0.9
174	Ovarian		37	Censored	Yes	4.2	1.1	2.8	0.3
175	Ovarian		59	Deceased	Yes	34.4	3.4	29.7	0.6
176	Ovarian		50	Censored	Yes	4.8	0.7	3.8	0.3
177	Ovarian		44	Deceased	Yes	5.4	1.4	3.4	0.5
178	Ovarian		50	Deceased	Yes	8.3	1.2	6.5	0.6
179	Ovarian		55	Deceased	Yes	3.6	1.1	2.4	0.1
180	Ovarian		35	Deceased	Yes	7.4	1.4	5.6	0.4
181	Ovarian		64	Deceased	Yes	15.2	1.7	12.6	0.7

182	Ovarian		61	Deceased	Yes	18.4	1.5	15.9	0.9
183	Ovarian		69	Censored	Yes	6.0	1.6	4.1	0.6
184	Ovarian		36	Deceased	Yes	6.7	1.9	4.4	0.4
185	Ovarian		61	Deceased	Yes	8.1	3.5	3.9	0.8
186	Ovarian		47	Deceased	Yes	6.9	2.3	4.4	0.2
187	Ovarian		71	Deceased	Yes	15.0	1.7	12.4	0.9
188	Ovarian		82	Deceased	Yes	24.5	2.1	21.1	1.3
189	Ovarian		71	Deceased	Yes	4.5	1.5	2.7	0.2
190	Ovarian		68	Deceased	Yes	4.6	0.8	3.5	0.4
191	Ovarian		67	Censored	Yes	5.7	1.0	4.5	0.2
192	Ovarian		61	Deceased	Yes	7.2	1.6	5.2	0.4
193	Ovarian		58	Deceased	Yes	5.5	1.2	4.8	0.2
194	Ovarian		63	Deceased	Yes	11.5	1.9	9.4	0.2
195	Ovarian		45	Still Living	Yes	2.1	1.2	1.3	0.1
196	Ovarian		63	Deceased	Yes	11.8	3.1	7.8	0.3
197	Ovarian		65	Deceased	Yes	8.2	1.7	5.7	0.7
198	Ovarian		55	Deceased	Yes	9.8	1.9	7.2	0.6
199	Ovarian		60	Deceased	Yes	5.0	1.3	3.7	0.2
200	Ovarian		45	Still Living	Yes	7.5	3.2	3.9	0.4
201	Ovarian		49	Deceased	Yes	3.2	1.1	2.1	0.3
202	Pancreatic	F	83	Deceased	No	7.6	1.2	4.4	0.6
203	Pancreatic	F	54	Deceased	No	5.5	1.7	3.5	0.3
204	Pancreatic	M	65	Deceased	No	9.5	1.6	7.1	0.8
205	Pancreatic	F	61	Deceased	No	7.9	1.6	5.8	0.5
206	Pancreatic	M	80	Censored	No	7.7	1.8	5.4	0.5
207	Pancreatic	M	73	Deceased	No	9.3	1.2	7.5	0.8
208	Pancreatic	M	70	Still Living	No	10.1	1.6	8	0.5
209	Pancreatic	M	73	Deceased	No	16.9	1.5	14.5	0.9
210	Pancreatic	M	67	Deceased	No	5.6	1.2	4.1	0.3
211	Pancreatic	F	64	Deceased	No	7.5	1.9	5.2	0.4
212	Pancreatic	M	72	Deceased	No	6.1	1.2	4.5	0.4
213	Pancreatic	M	59	Deceased	No	8.2	1.6	6.2	0.5
214	Pancreatic	M	74	Deceased	No	13.7	1.4	11.4	0.9
215	Pancreatic	M	67	Deceased	No	11.4	2.8	7.7	0.9
216	Pancreatic	F	70	Censored	No	15.0	3.7	10.9	0.4
217	Pancreatic	F	71	Deceased	No	4.3	1.6	2.3	0.4
218	Pancreatic	M	79	Deceased	No	6.3	1.1	4.7	0.5
219	Pancreatic	M	52	Deceased	No	9.8	2.2	7.4	0.2
220	Pancreatic	M	57	Deceased	No	9.0	1.4	6.9	0.7
221	Pancreatic	F	60	Deceased	No	7.4	2.8	4.4	0.2
222	Pancreatic	M	81	Deceased	No	15.0	0.7	13.9	0.4
223	Pancreatic	M	72	Censored	No	11.6	1.6	9.9	0.1
224	Pancreatic	M	62	Deceased	No	12.6	1.7	10.5	1.1
225	Pancreatic	M	72	Deceased	No	6.4	1.3	4.5	0.6
226	Pancreatic	M	53	Deceased	No	11.4	2.8	7.8	0.7
227	Pancreatic	M	73	Still Living	No	7.6	1.1	6.2	0.2

228	Pancreatic	M	61	Deceased	No	11.2	1.6	8.7	0.9
229	Pancreatic	F	78	Deceased	No	8.6	2.5	5.7	0.4
230	Pancreatic	M	70	Censored	No	15.3	1.4	13.2	0.8
231	Pancreatic	F	66	Deceased	No	4.3	1.3	2.7	0.3
232	Pancreatic	M	51	Deceased	No	4.4	1.2	2.7	0.5
233	Pancreatic	F	66	Censored	No	8.2	2.0	5.6	0.6
234	Pancreatic	F	65	Deceased	No	6.5	1.9	4.2	0.4
235	Pancreatic	F	75	Still Living	No	8.3	1.4	6.3	0.6
236	Pancreatic	F	69	Deceased	No	5.4	1.6	3.5	0.3
237	Pancreatic	M	63	Deceased	No	8.3	2.2	4.6	0.9
238	Pancreatic	M	75	Deceased	No	10.9	1.8	8.5	0.5
239	Pancreatic	M	81	Censored	No	10.8	2.0	8.4	0.4
240	Pancreatic	M	64	Deceased	No	5.6	1.3	3.8	0.5
241	Pancreatic	F	77	Deceased	No	15.4	1.3	13.9	0.2
242	Pancreatic	M	58	Deceased	No	10.0	0.9	8.7	0.4
243	Pancreatic	M	39	Deceased	No	7.7	1.2	6.3	0.3
244	Pancreatic	M	50	Deceased	No	6.9	1.9	4.6	0.5
245	Pancreatic	M	84	Censored	No	7.2	1.4	5.5	0.3
246	Pancreatic	F	68	Still Living	No	4.1	0.8	3.0	0.3
247	Pancreatic	M	63	Deceased	No	7.4	2.8	3.9	0.7
248	Pancreatic	F	50	Censored	No	14.2	3.1	10.2	0.9
249	Pancreatic	F	65	Censored	No	5.6	2.0	3.3	0.3
250	Pancreatic	F	63	Deceased	No	9.1	1.8	6.9	0.4
251	Pancreatic	F	64	Still Living	No	3.9	1.2	2.4	0.3
252	Pancreatic	M	85	Censored	No	6.6	1.4	4.5	0.7
253	Pancreatic	M	61	Deceased	No	10.2	1.9	7.7	0.6
254	Pancreatic	M	59	Deceased	No	9.5	1.0	7.8	0.7
255	Pancreatic	M	60	Deceased	Yes	6.4	1.8	4.5	0.1
256	Pancreatic	M	66	Censored	Yes	6.3	1.7	3.7	0.6
257	Pancreatic	F	71	Deceased	Yes	7.6	1.4	5.8	0.4
258	Pancreatic	F	76	Censored	Yes	9.5	0.7	8.3	0.6
259	Pancreatic	M	83	Deceased	Yes	10.4	2.0	8.1	0.3
260	Pancreatic	F	76	Deceased	Yes	16.9	1.6	14.7	0.6
261	Pancreatic	F	77	Censored	Yes	7.1	1.2	5.3	0.6
262	Pancreatic	F	69	Deceased	Yes	8.8	2.2	6.0	0.6
263	Pancreatic	M	42	Deceased	Yes	7.0	1.2	5.2	0.5
264	Pancreatic	M	77	Deceased	Yes	5.1	2.3	3.4	1.0
265	Pancreatic	M	67	Deceased	Yes	4.4	1.0	3.2	0.2
266	Pancreatic	M	69	Censored	Yes	2.9	0.9	1.7	0.3
267	Pancreatic	F	79	Still Living	Yes	8.7	2.3	6.0	0.3
268	Pancreatic	M	42	Deceased	Yes	73.3	1.5	71.7	0.1
269	Pancreatic	F	83	Still Living	Yes	8.1	2.7	5.0	0.4
270	Pancreatic	F	50	Deceased	Yes	3.3	1.1	1.9	0.3
271	Pancreatic	F	64	Censored	Yes	6.9	0.9	5.5	0.4
272	Pancreatic	M	62	Deceased	Yes	12.5	0.9	8.6	1.2
273	Pancreatic	M	79	Deceased	Yes	9.5	3.6	5.5	0.5
274	Pancreatic	M	74	Deceased	Yes	7.4	1.1	5.8	0.4

275	Pancreatic	M	62	Deceased	Yes	6.0	1.7	4.2	0.1
276	Pancreatic	F	60	Deceased	Yes	7.9	1.4	5.9	0.6
277	Pancreatic	F	58	Deceased	Yes	6.4	0.8	4.7	0.5
278	Pancreatic	F	61	Still Living	Yes	3.3	0.9	2.2	0.1
279	Pancreatic	F	41	Deceased	Yes	8.6	1.9	6.1	0.6
280	Pancreatic	M	64	Still Living	Yes	4.8	1.6	2.9	0.4
281	Pancreatic	M	64	Censored	Yes	8.2	1.0	6.7	0.5
282	Pancreatic	F	67	Deceased	Yes	8.3	0.6	7.4	0.3
283	Pancreatic	F	51	Deceased	Yes	7.1	0.9	5.9	0.3
284	Pancreatic	F	63	Deceased	Yes	4.5	2	2.2	0.4
285	Pancreatic	F	73	Deceased	Yes	4.6	1.9	2.2	0.5
286	Pancreatic	M	63	Deceased	Yes	7.1	0.4	6.6	0.1
287	Pancreatic	F	69	Deceased	Yes	5.9	0.8	4.9	0.2
288	Pancreatic	F	73	Deceased	Yes	6.3	1.2	4.7	0.4
289	Pancreatic	M	68	Censored	Yes	8.3	0.6	7.5	0.2
290	Pancreatic	F	61	Deceased	Yes	4.3	0.4	3.9	0.1
291	Pancreatic	F	51	Deceased	Yes	4.4	1.4	2.7	0.2
292	Pancreatic	M	66	Deceased	Yes	9.2	2.2	6.3	0.3
293	Pancreatic	F	66	Censored	Yes	6.1	1.1	4.7	0.3
294	Pancreatic	M	64	Deceased	Yes	7.6	1.6	5.6	0.4
295	Pancreatic	F	55	Censored	Yes	10.9	1.3	9.2	0.4
296	Pancreatic	M	71	Deceased	Yes	9.3	2	6.8	0.5
297	Pancreatic	M	47	Censored	Yes	6.0	1.9	3.7	0.4
298	Pancreatic	M	65	Deceased	Yes	15.4	1.3	12.6	1.5
299	Pancreatic	M	78	Censored	Yes	23.9	1.8	21.2	0.9
300	Pancreatic	F	74	Censored	Yes	6.9	2.0	4.1	0.8
301	Pancreatic	F	84	Censored	Yes	9.9	2.5	7.0	0.3
302	Pancreatic	M	60	Deceased	Yes	7.3	1.0	5.8	0.5
303	Pancreatic	F	52	Deceased	Yes	26.5	3.3	22.2	1.0
304	Pancreatic	M	61	Deceased	Yes	9.2	1.8	7.1	0.3
305	Pancreatic	F	67	Deceased	Yes	3.9	1	2.8	0.2
306	Pancreatic	F	75	Censored	Yes	6.5	0.6	5.4	0.5
307	Pancreatic	M	70	Censored	Yes	5.1	1.2	3.7	0.2

HGB	HCT	PLT	Albumin	Alk_Phos	Bilirubin	LMR	NLR	TP	AS
11.0	34.7	362.0	3.4	461.0	0.7	1.9	4.7	7.4	4
11.7	34.7	246.0	4.0	100.0	0.4	1.9	2.4	6.5	1
9.2	28.3	184.0	3.4	578.0	1.0	1.9	1.7	6.3	2
8.7	28.5	708.0	1.2	67.0	0.5	1.5	7.4	6.7	4
11.4	34.3	289.0	3.1	498.0	8.5	2.1	4.3	6.8	3
11.0	37.6	246.0	2.1	495.0	0.7	0.9	6.5	5.4	3
15.4	46.0	210.0	4.2	199.0	1.3	2.0	2.9	6.9	1
11.9	36.9	480.0	3.8	374.0	3.4	2.8	5.3	7.1	1
11.5	36.0	321.0	3.5	456.0	2.8	1.0	8.8	6.3	3
14.1	42.0	517.0	4.3	59.0	0.4	10.0	2.6	7.1	0
14.0	42.7	343.0	10.7	493.0	10.9	2.6	6.5	6.1	2
15.3	49.1	188.0	4.6	92.0	0.9	10.3	1.5	7.5	0
13.2	40.8	371.0	4.0	169.0	0.5	7.3	3.1	6.5	1
13.8	41.2	127.0	4.2	83.0	0.2	5.5	1.0	7.7	0
11.4	34.4	103.0	3.8	188.0	1.0	6.5	8.3	7.0	2
11.1	32.5	347.0	4.0	253.0	1.7	3.7	1.9	7.0	0
14.9	43.5	125.0	4.5	50.0	0.7	3.3	4.2	7.0	1
10.5	31.0	152.0	4.0	43.0	0.6	3.3	3.0	6.2	0
11.1	35.7	228.0	4.3	158.0	0.3	3.8	1.6	7.2	0
12.7	38.5	87.0	4.2	84.0	0.5	2.4	2.3	7.1	0
9.6	29.7	189.0	3.3	421.0	2.1	2.5	5.1	6.0	2
10.8	36.6	204.0	3.5	185.0	0.7	2.1	6.1	6.1	2
11.4	35.0	86.0	2.3	482.0	5.2	1.3	5.7	6.3	3
10.9	31.8	179.0	5.2	129.0	1.0	2.6	4.2	7.1	1
10.1	29.3	58.0	4.2	228.0	0.3	3.0	11.4	7.5	2
11.8	38.1	427.0	4.1	97.0	0.6	3.0	3.8	6.8	1
11.7	36.5	101.0	3.7	60.0	0.9	1.8	4.1	6.9	2
13.6	40.1	123.0	4.0	59.0	0.4	8.5	2.4	6.1	0
12.8	41.5	309.0	4.5	261.0	0.5	3.5	5.9	8.3	1
12.9	40.4	157.0	3.7	69.0	0.6	3.0	2.4	6.7	0
12.0	36.1	97.0	2.5	248.0	1.6	1.5	11.1	5.7	4
13.6	41.3	205.0	4.1	64.0	0.8	7.3	1.8	6.7	0
9.8	31.1	277.0	3.5	257.0	2.3	4.0	2.4	6.4	0
9.4	28.9	135.0	1.6	93.0	0.4	2.2	8.3	8.1	3
11.1	34.1	254.0	4.0	154.0	0.7	4.0	1.4	7.5	0
12.5	39.6	354.0	4.3	142.0	0.5	3.5	4.9	7.8	1
8.6	27.4	502.0	3.7	68.0	0.2	2.3	3.6	6.7	1
11.6	38.3	194.0	4.2	116.0	0.3	4.8	1.8	7.4	0
10.7	33.9	169.0	2.9	244.0	0.4	16.0	3.5	6.8	3
9.9	32.2	364.0	4.1	126.0	0.3	4.0	3.8	7.5	1
12.8	39.6	216.0	4.0	80.0	0.6	6.7	3.7	6.7	1
12.1	38.2	452.0	3.9	108.0	0.3	3.7	2.7	6.7	0
12.3	37.6	384.0	3.9	90.0	0.4	2.9	2.1	7.2	0
10.8	34.7	406.0	4.2	86.0	0.2	4.3	3.9	6.8	1
14.9	42.0	180.0	4.4	66.0	0.9	6.7	3.2	7.4	1

14.0	42.8	213.0	3.6	131.0	0.8	4.0	4.9	7.8	1
12.2	37.9	197.0	4.0	281.0	1.2	2.2	5.8	7.1	1
11.5	35.1	149.0	4.1	90.0	0.2	5.5	2.2	6.9	0
12.0	36.4	373.0	3.6	118.0	0.3	2.3	4.6	6.3	1
11.4	35.6	448.0	2.7	186.0	0.6	3.4	6.1	6.3	3
15.1	46.1	191.0	5.0	43.0	0.9	22.0	1.4	7.3	0
11.5	37.1	350.0	3.8	103.0	0.4	4.3	4.7	6.9	1
14.2	44.5	176.0	5.0	72.0	0.4	7.7	1.3	7.2	0
12.3	37.5	132.0	4.0	130.0	0.6	3.0	3.0	6.8	0
13.2	39.8	284.0	3.9	188.0	0.4	2.8	1.8	6.9	0
13.0	39.7	178.0	4.2	97.0	0.7	7.0	1.7	6.9	0
10.1	32.3	251.0	3.1	54.0	0.2	6.5	5.0	6.1	2
12.5	37.4	313.0	3.8	69.0	0.5	2.0	7.8	6.9	2
12.1	38.1	285.0	2.5	830.0	4.6	2.0	6.2	6.8	3
9.9	29.8	73.0	2.6	615.0	1.3	3.3	6.2	5.8	3
11.1	34.9	193.0	4.0	171.0	0.3	5.4	1.6	7.5	0
12.0	37.1	409.0	4.5	115.0	0.1	2.5	2.7	7.7	0
11.0	36.7	209.0	2.5	465.0	7.7	0.9	7.9	6.2	3
11.1	34.5	228.0	4.1	560.0	0.5	1.3	5.4	7.2	2
11.5	36.8	433.0	3.6	345.0	0.8	3.9	2.1	8.9	0
12.7	40.9	267.0	4.4	137.0	0.5	2.8	5.0	7.6	1
13.8	41.9	241.0	3.5	138.0	1.1	3.2	2.7	6.8	0
13.0	38.7	225.0	3.9	167.0	0.7	2.2	3.2	7.1	1
15.1	45.5	209.0	4.4	71.0	0.4	12.0	5.1	7	1
16.0	47.5	212.0	4.7	122.0	0.5	7.0	4.2	7.4	2
11.2	36.0	193.0	3.6	135.0	0.5	3.0	4.0	6.4	1
10.1	30.6	389.0	2.9	653.0	1.4	2.0	4.3	6.9	3
8.5	26.5	666.0	2.6	120.0	0.4	4.8	6.4	5.5	3
12.0	36.5	262.0	3.8	75.0	0.5	3.3	4.2	6.5	1
11.5	36.4	282.0	3.9	175.0	0.3	2.0	2.8	7.5	1
9.8	31.6	83.0	3.6	382.0	0.4	3.3	2.5	6.2	0
10.8	33.3	543.0	2.8	366.0	2.2	13.0	6.2	6.2	3
9.9	31.7	490.0	2.7	125.0	0.6	3.0	3.2	6.1	2
11.9	36.3	252.0	4.2	63.0	0.4	3.3	3.0	6.8	0
12.8	39.1	285.0	3.9	231.0	0.6	2.8	2.6	6.6	0
13.4	43.1	273.0	3.5	70.0	0.2	6.5	2.3	6.3	0
9.9	32.7	257.0	3.9	186.0	0.4	3.2	0.6	7.6	0
11.6	34.9	104.0	3.5	122.0	0.6	1.7	5.1	6.7	3
11.8	36.7	295.0	4.0	55.0	0.6	2.5	6.0	6.5	1
7.0	21.7	503.0	3.8	86.0	0.2	3.6	3.7	6.1	1
12.8	39.3	113.0	3.3	260.0	1.1	3.0	3.1	6.3	2
12.0	35.4	115.0	3.7	257.0	0.7	2.4	4.0	6.9	1
13.0	39.5	340.0	3.5	175.0	0.5	2.8	3.4	5.4	1

11.4	35.9	218.0	3.5	112.0	0.7	5.7	2.1	6.8	0
11.4	35.1	193.0	3.2	201.0	0.6	2.0	3.9	6.5	3
12.1	39.0	375.0	4.2	133.0	0.5	5.7	3.6	6.9	1
12.5	36.2	251.0	3.8	485.0	0.3	2.2	3.1	6.1	1
14.0	42.7	297.0	3.8	280.0	0.3	11.0	3.0	7.5	0
10.1	31.2	217.0	3.8	182.0	1	2.3	3.7	7.1	1
12.3	37.8	467.0	4.2	96.0	0.3	5.0	3.4	7.5	1
12.5	37.4	264.0	4.2	116.0	0.5	3.9	1.9	7.4	0
14.4	41.9	234.0	4.5	71.0	0.6	11.0	3.0	6.9	1
11.9	38.9	211.0	4.0	67.0	0.4	4.0	1.9	6.7	0
12.8	39.6	215.0	4.2	130.0	0.3	2.6	2.4	7.2	0
10.0	32.3	250.0	4.0	65.0	0.3	3.6	1.8	7.1	0
13.6	42.3	213.0	4.0	86.0	0.4	3.4	2.8	7.9	0
11.2	33.6	139.0	2.9	127.0	0.7	14.0	23.6	5.2	3
8.4	28.1	223	3.8	54	0.5	5.0	5.6	6.6	1
12.3	37.6	259.0	4.3	60.0	0.8	10.0	3.1	6.6	1
11.8	36	264	4.3	589	1.3	3.5	2.0	6.8	0
10.1	31.2	136.0	3.9	146.0	0.3	5.0	8.2	6.5	1
13.0	39.1	477.0	4.0	47.0	0.7	5.3	3.6	6.9	1
12.3	36.0	259.0	4.4	45.0	0.5	5.3	2.4	7.3	0
11.9	37.7	395.0	4.5	82.0	0.3	6.3	2.5	7.1	0
11.5	35.1	176.0	1.5	841.0	0.7	1.9	8.1	4.7	4
13.1	42.4	332	3	85	0.6	2.5	1.6	5.6	1
12.8	39.3	266.0	4.7	39.0	0.3	8.0	1.1	7.5	0
11.3	35.5	334.0	4.6	96.0	0.3	4.7	1.4	7.5	0
9.8	29.5	171	3.8	144	0.6	4.0	2.6	6.3	0
10.7	32.7	157.0	3.7	137.0	0.3	2.5	2.7	7.3	0
14.0	43.4	260.0	3.9	86.0	0.2	3.6	2.4	6.9	0
6.5	21.2	526.0	2.5	110.0	0.3	1.5	3.9	6	3
10.4	31.7	400.0	4.1	53.0	0.3	3.2	2.3	7	0
9.4	29.8	452.0	3.1	214.0	0.2	3.0	3.2	6.9	2
13.6	41.8	278.0	3.8	68.0	0.5	22.0	2.7	6	0
9.6	30.5	363.0	4.0	51.0	0.3	5.3	2.8	6.7	0
10.7	33.3	410.0	3.6	208.0	0.4	3.2	3.1	6.8	1
11.8	34.7	183.0	4.2	105.0	0.3	3.0	2.3	6.8	0
11	34.5	379	4.1	61	0.4	2.3	6.4	6.4	1
10.1	31.1	268.0	3.6	234.0	0.4	3.3	13.6	6.2	2
9.4	30	206	3.6	122	0.4	3.2	1.7	6.4	0
12.9	41.5	237.0	4.2	65.0	0.4	7.5	1.9	6.6	0
13.7	43.2	560.0	2.9	191.0	0.3	4.0	8.6	6	3
10.0	31.7	473.0	3.7	59.0	0.4	8.0	3.8	6.8	1
9.9	29.5	586.0	3.3	878.0	1.9	2.5	4.0	5.6	2
9.9	31.0	53.0	3.9	105.0	0.4	1.8	15.1	6	3
9.5	28.7	227	4	110	0.6	2.0	3.5	6.6	2
12.2	36.6	275.0	3.8	80.0	0.3	1.3	3.6	6.4	2
12.9	40.4	204.0	4.0	82.0	0.5	3.1	2.4	7.1	0

11.8	38.5	353	4.4	90	0.3	4.0	2.9	7.2	0
11.5	36.3	148.0	3.7	92.0	0.9	5.0	3.9	6.6	1
10.1	30.4	202.0	4.2	77.0	0.3	3.5	1.9	7	0
10.4	32.7	204.0	4.1	121.0	0.3	6.0	1.9	7.6	0
12.3	37.3	425.0	4.2	95.0	0.3	4.0	0.9	7.3	0
12.8	38.6	314.0	3.5	90.0	0.2	3.3	2.5	6.2	0
10.8	32.3	288.0	3.6	236.0	0.6	4.2	0.8	6.5	0
8.7	26.6	802.0	2.1	1194.0	5.3	1.3	6.4	5.2	4
12.5	36.8	186.0	4.6	77.0	0.3	5.2	1.2	7	0
10.9	33.2	161.0	4.5	189.0	0.5	6.5	4.8	6.9	1
10.6	32.6	822.0	3.4	551.0	10.3	2.5	5.5	6.5	2
8.5	27.6	97.0	3.1	87.0	0.7	3.7	4.5	6.2	2
12.9	39.0	291.0	4.5	90.0	0.3	3.5	2.1	7.8	0
12.1	37.0	182.0	4.2	68.0	0.9	2.7	2.9	6.8	0
11.0	33.3	174.0	4.2	124.0	0.3	2.2	6.2	7	2
14.4	44.1	246.0	4.5	87.0	0.4	8.7	2.8	6.9	0
9.7	31.4	219.0	4.4	62.0	0.4	3.7	3.4	6.7	1
12.4	39.4	276	4.2	832	1.9	3.1	3.3	7.7	1
12.0	36.8	526.0	4.1	129.0	0.3	2.0	2.4	7.1	2
10.9	34.3	295.0	4.0	84.0	0.3	5.0	3.3	7	1
11.7	35.5	224.0	4.6	102.0	0.4	2.0	4.4	6.7	2
10.4	34.7	647	3	380	0.3	11.5	3.5	5.6	2
11.9	35.8	154.0	4.6	96.0	0.3	3.8	2.0	6.7	0
10.3	31.0	334.0	3.4	549.0	0.5	3.0	6.5	5.6	3
11	33.1	144	3.8	78	0.4	10.0	1.5	7.2	0
8.9	26.6	67	3.4	403	0.6	9.0	2.2	6.4	1
10.3	31.9	394.0	3.8	110.0	0.4	1.7	4.8	6.8	3
13.1	41.2	216.0	4.3	125.0	0.9	3.0	2.5	8.3	0
8	24.7	124	3.3	52	0.8	6.0	2.7	5.6	1
11.6	34.4	284.0	4.3	77.0	0.4	6.8	2.3	6.8	0
9.4	28.6	204.0	4.0	76.0	0.2	3.5	2.2	6.7	0
10.4	32.6	277	3.6	98	0.3	4.6	1.7	6.4	0
12.8	37.5	342.0	4.4	56.0	0.4	5.0	3.7	6.8	1
10.9	36.0	169.0	3.9	62.0	0.3	3.7	2.5	7.3	0
13.8	41.2	162.0	4.1	89.0	0.3	4.0	0.6	6.3	0
12.6	39.5	245.0	4.3	58.0	0.4	6.0	3.1	6.9	1
8.7	27.6	354	3	551	1.3	2.2	6.5	7.2	3
12.0	36.4	233.0	3.9	94.0	0.2	2.3	4.3	6.5	1
8.7	28.5	408.0	3.1	86.0	0.3	1.9	5.6	6.4	4
12.7	42.2	242.0	4.8	54.0	0.8	3.7	2.5	7	0
9.8	31.3	47.0	3.8	163.0	0.2	5.7	8.7	6.4	2
11.4	33.9	133.0	2.7	121.0	2.2	2.3	5.4	5.6	2
13.1	38.8	265.0	4.1	91.0	0.3	2.8	2.4	6.8	0
10.8	31.7	433.0	3.4	160.0	0.6	2.0	5.4	7.9	3
12.1	35.4	83.0	4.4	52.0	0.5	11.0	2.2	6.4	0
10.9	34.1	293.0	4.2	96.0	0.3	3.5	4.0	7.9	1
10.3	32.1	646.0	3.1	1154.0	0.6	2.4	7.4	6.9	3

8.4	26.4	708.0	2.4	151.0	0.3	1.7	10.6	5.4	4
11.3	32.4	155.0	4.1	107.0	0.4	2.7	2.6	6.9	0
9.7	30.3	614.0	3.9	85.0	0.2	4.8	2.3	7.2	0
10.8	33.8	508.0	4.0	71.0	0.5	4.4	1.1	7.3	0
12.0	37.4	308.0	4.6	50.0	0.3	11.5	1.9	7.2	0
11.1	32.6	491.0	2.9	72.0	0.2	1.9	7.3	5.3	4
9.3	28.6	331	3.7	124	0.4	1.6	10.0	6.7	3
11.5	35.2	290.0	4.3	120.0	0.6	7.5	1.8	7.4	0
9.9	30.0	343.0	3.7	71.0	0.3	2.0	4.4	6.5	2
13.2	39.9	309.0	4.4	69.0	0.5	5.0	4.5	6.6	1
14.3	41.1	179.0	4.0	78.0	0.5	4.0	3.3	6.4	1
10.3	34.2	276.0	4.0	156.0	0.3	6.0	4.0	7.6	1
10.3	34.1	689	3.8	117	0.4	9.5	4.9	6.2	2
11.2	33.6	109.0	4.5	89.0	0.5	12.0	1.1	6.8	0
10.1	30.8	303.0	4.6	215.0	0.3	10.3	2.5	7	0
11.9	37.7	188.0	4.1	130.0	0.2	2.4	3.4	7.1	1
11.3	34.5	342.0	4.2	41.0	0.5	3.2	3.8	6.5	1
13.4	39.4	287.0	4.2	81.0	0.3	6.5	2.8	6.7	0
13.2	40.7	253	4.5	89	0.5	8.0	1.2	7.2	0
8.2	26.1	237.0	4.1	100.0	0.3	3.7	1.9	7.5	0
11.3	35.8	282.0	4.5	63.0	0.4	2.0	3.7	6.8	2
11.5	35.8	230.0	4.1	50.0	0.4	5.7	2.1	6.6	0
13.6	40	158	3.2	83	0.5	2.0	4.4	7.1	3
12.7	37.9	312.0	3.5	93.0	0.4	3.2	3.6	6.1	1
10.9	32.5	218.0	3.8	90.0	0.7	3.6	3.0	6.7	0
10	29.8	313	2.9	109	3.9	1.5	6.3	5.7	3
15.7	48.4	166	4.1	89	0.5	3.2	5.0	6.3	1
11.4	37.5	351.0	3.0	108.0	0.6	1.7	9.7	5.9	4
9.0	26.9	172.0	3.5	54.0	0.8	4.0	3.4	6.2	1
14.0	42.1	275.0	4.0	69.0	0.5	4.8	2.7	7.5	0
14.2	41.2	66.0	3.7	71.0	1.6	3.0	3.8	6.7	1
14.2	43.7	182	4.1	326	3.8	3.2	3.9	5.7	1
13.2	40.6	487	3.7	465	0.7	1.6	8.1	6.1	3
14	41.9	257	4.2	297	1.1	3.1	2.8	7.0	0
10.4	33.8	308.0	2.3	1087.0	1.1	9.3	2.9	5.3	2
12.2	37.4	229.0	3.6	108.0	0.4	4.0	1.4	6.7	0
13.5	42.4	138	3.9	139	0.8	2.2	4.3	6.5	1
14.3	43.3	331	4.6	47	0.6	11.0	3.4	6.6	1
15.2	46.6	311.0	4.4	160.0	0.8	2.0	4.9	6.9	2
14.2	45.1	293	4.1	364	0.3	14.0	1.6	7.2	0
10.8	32.8	223.0	3.3	201.0	17.7	1.8	19.9	5.6	4
12.9	41.0	407.0	4.1	238.0	0.5	16.0	6.2	7.0	2
14.4	43.2	277.0	3.4	209.0	0.9	1.5	6.2	5.8	4
11.7	36.1	162	3.4	192	0.7	2.2	3.5	5.8	2
14.3	47.3	307.0	4.4	70.0	0.4	4.0	2.8	6.9	0
12.8	38.3	272.0	4.4	44.0	0.6	5.5	5.6	7.2	1

11.2	35.1	169	3.8	197	1.1	1.8	5.4	6.4	3
14.4	45.6	256.0	3.8	74.0	0.6	6.3	2.3	6.3	0
15.0	46.2	279.0	3.4	317.0	1.0	1.8	9.4	5.6	4
10.9	33.5	353.0	3.8	94.0	0.7	4.3	2.1	6.0	0
11.1	35.0	209.0	3.8	92.0	0.6	2.4	2.3	6.2	0
12.9	40.4	324.0	4.1	137.0	6.4	3.3	2.8	7.3	0
11.5	36.7	185.0	4.1	105.0	0.3	4.8	2.2	6.7	0
12.7	39.1	247.0	3.7	143.0	0.5	2.3	4.5	6.6	1
13.3	41.5	337.0	4.0	42.0	0.6	5.3	2.2	6.3	0
14.6	43.3	279	3.1	84	1.1	2.4	2.1	7.2	1
11.1	35.5	218	2.9	116	1.1	3.6	4.7	5.9	3
13.7	42.1	224.0	4.1	155.0	1.9	5.0	4.2	5.7	2
14.2	41.5	270.0	4.0	50.0	0.5	2.6	2.9	6.3	0
11.4	4.9	527	3.4	86	0.3	6.5	10.7	6.1	3
9.9	30.2	307.0	2.7	167.0	9.3	2.3	9.7	4.7	3
14.7	44.1	193	4.1	284	2.1	4.0	5.3	6.5	1
11.4	35.9	377.0	3.7	116.0	0.2	3.8	2.4	6.0	0
10.6	33.2	416.0	3.6	97.0	0.3	4.7	3.9	7.0	1
9.6	30.8	172.0	4.2	88.0	0.5	2.7	3.8	7.2	1
15.1	43.2	147	4.2	110	0.7	4.0	1.4	7.8	0
10.4	34.4	475.0	3.4	76.0	0.2	3.4	3.3	7.0	3
11.7	35.9	204.0	3.7	84.0	0.6	6.7	1.7	5.7	0
12.3	37.2	359.0	3.9	163.0	1.1	4.5	3.8	6.6	1
11.3	35.3	208.0	3.7	72.0	1.2	4.0	2.0	6.2	0
12.9	38.8	370.0	4.1	469.0	0.6	2.0	3.2	7.7	2
15.9	49.6	252	2.6	158	0.5	3.2	4.1	7.4	2.0
13.9	41.4	344.0	4.3	308.0	6.6	1.4	7.8	6.5	2.0
11.6	37.6	387.0	3.9	68.0	0.4	18.0	2.5	8.1	0
10.8	33.0	183.0	3.5	93.0	0.6	2.8	2.2	6.1	0
11.1	33.4	157.0	4.2	114.0	0.2	3.5	4.1	6.9	1
9.6	31.4	406.0	3.2	142.0	1.1	1.2	11.9	5.7	4
11.6	35.7	286.0	2.6	190.0	1.0	6.7	4.1	5.7	3
10.9	34.4	412.0	4.3	445.0	1.0	2.7	9.2	6.8	2
7.9	26.8	282.0	4.4	227.0	0.3	2.0	4.4	7.5	2
9.5	30.5	360.0	3.3	342.0	0.4	3.7	2.7	5.8	1
13.8	41.2	146	4.4	72	0.5	2.4	4.3	7.2	1
14.7	45.6	158.0	3.9	56.0	0.7	2.3	1.5	5.9	0
11.4	36.1	104.0	3.8	212.0	1.7	5.0	3.2	6.6	1
9.7	30.8	218.0	3.4	74.0	0.6	3.0	1.9	6.8	1
10.0	31.1	244.0	4.0	84.0	0.3	7.7	2.6	6.8	0
12.3	37.1	224.0	4.5	192.0	0.4	15.0	47.8	7.3	2
11.5	35.6	210.0	4.2	90.0	0.4	6.8	1.9	7.2	0
10.9	32.2	330	3.5	341	7.6	3.7	1.7	5.5	0
10.2	31.9	136.0	4.0	607.0	0.6	2.3	6.1	6.4	1
8.7	27.8	407.0	3.3	156.0	0.3	0.8	9.6	5.4	4
14.6	45.3	220	3.5	161	0.6	7.2	1.5	7.2	0
12.7	40.6	241	3.4	227	0.4	2.8	5.3	6.5	2

9.4	30.8	445	3.5	70	0.3	17.0	2.5	7.2	0
11.2	36.3	283	3.9	125	0.4	2.3	4.2	6.0	1
9.8	30.4	188.0	3.5	429.0	0.6	1.6	5.9	6.3	2
12	35.1	137	4	61	0.5	9.0	2.4	6.4	0
12.3	38.4	89.0	4.8	132.0	0.4	3.2	3.2	7.7	1
11.7	35.1	180	4.7	114	0.7	4.0	1.8	6.6	0
9.2	28.0	453.0	2.8	491.0	6.0	2.0	6.7	6.3	3
12.2	36.9	312.0	3.2	1001.0	1.5	2.0	12.3	6.4	3
11.1	36.0	303.0	3.4	146.0	0.5	3.0	6.6	7.4	2
9.4	29.9	325	4.1	138	0.3	5.0	1.1	6.3	0
10.5	34.7	23.7	3.8	78.0	0.7	3.8	1.2	6.9	0
7.9	24.7	210.0	2.9	80.0	0.6	4.0	16.5	5.1	2
10.3	32.6	147.0	4.0	280.0	0.5	4.0	6.1	6.0	1
11.3	34.9	178.0	3.6	98.0	0.4	3.0	3.9	6.5	1
11.6	33.3	160.0	3.2	112.0	0.9	3.0	12.5	5.7	2
8.6	24.3	81.0	3.7	603.0	1.3	4.0	9.8	6.2	1
12.5	40.4	307.0	4.5	81.0	0.3	7.0	1.9	6.8	0
6.7	22.4	343	3.2	250	0.7	7.3	2.9	7.0	1
11.8	36.6	143.0	3.9	61.0	0.4	3.7	4.3	6.0	1
13.4	40.4	198.0	4.8	61.0	0.4	4.0	3.5	7.4	1
8.3	25.8	182.0	3.3	911.0	1.0	3.3	7.1	5.9	3
10.9	34.8	376	3.9	200	0.3	4.0	3.4	7.0	1.0
13.5	40.5	168.0	4.3	50.0	0.2	4.8	1.9	6.2	0.0
14.2	44.6	366.0	2.5	1190.0	3.3	0.9	9.7	5.5	4
9.7	29.5	390.0	3.6	116.0	0.8	2.0	11.8	5.8	3
13.1	40.9	182.0	4.6	118.0	0.4	2.5	2.1	7.1	0.0
12.9	40.2	347.0	4.2	99.0	2.2	8.3	2.8	6.6	0.0
8.9	27.6	224.0	2.9	164.0	0.5	2.0	5.8	5.4	3.0
8.9	27.5	212.0	2.8	207.0	1.1	3.3	6.7	5.5	3.0
9.0	28.8	181.0	3.6	63.0	0.5	6.0	3.9	6.0	1.0
9.9	30.6	234	3.5	161	0.5	5.0	2.8	7.1	0.0
13.0	40.0	273.0	4.5	100.0	0.6	1.2	9.0	7.2	2.0
9.1	29.1	240.0	3.9	69.0	0.4	6.0	3.1	6.2	1.0

Prog Fac	Surv (Days)	WBC	Hgb	LY
High Risk	518	11.8	11.0	1.9
Intermediate-Risk	3210	5.9	11.7	1.5
Intermediate-Risk	1610	4.2	9.2	1.3
High Risk	59	13.6	8.7	1.5
High Risk	84	12.2	11.4	2.1
High Risk	20	8.6	11.0	1.0
Intermediate-Risk	1455	8.2	15.4	1.8
Intermediate-Risk	1990	9.4	11.9	1.4
High Risk	674	14.1	11.5	1.3
Low-Risk	1144	7.3	14.1	2.0
Intermediate-Risk	100	18.2	14.0	2.3
Low-Risk	2761	7.3	15.3	3.1
Intermediate-Risk	200	9.3	13.2	2.2
Low-Risk	485	4.5	13.8	2.2
Intermediate-Risk	151	12.3	11.4	1.3
Low-Risk	77	7.0	11.1	2.2
Intermediate-Risk	1235	5.5	14.9	1.0
Low-Risk	610	4.3	10.5	1.0
Low-Risk	598	6.7	11.1	2.3
Low-Risk	766	6.3	12.7	1.7
Intermediate-Risk	85	6.6	9.6	1.0
Intermediate-Risk	77	11.4	10.8	1.5
High Risk	281	7.3	11.4	1.0
Intermediate-Risk	15	7.3	10.9	1.3
Intermediate-Risk	2547	19.1	10.1	1.5
Intermediate-Risk	281	11.5	11.8	2.1
Intermediate-Risk	268	4.0	11.7	0.7
Low-Risk	2323	5.9	13.6	1.7
Intermediate-Risk	431	5.0	12.8	0.7
Low-Risk	1820	6.8	12.9	1.8
High Risk	67	11.5	12.0	0.9
Low-Risk	1724	5.8	13.6	2.2
Low-Risk	1595	5.9	9.8	1.6
High Risk	47	10.6	9.4	1.1
Low-Risk	2144	4.3	11.1	1.6
Intermediate-Risk	265	8.7	12.5	1.4
Intermediate-Risk	548	7.9	8.6	1.6
Low-Risk	1685	5.7	11.6	1.9
High Risk	208	14.6	10.7	3.2
Intermediate-Risk	1051	6.0	9.9	1.2
Intermediate-Risk	546	9.6	12.8	2.0
Low-Risk	201	10.3	12.1	2.6
Low-Risk	2079	10.0	12.3	2.9
Intermediate-Risk	297	8.7	10.8	1.7
Intermediate-Risk	220	8.7	14.9	2.0

Intermediate-Risk	1891	10.0	14.0	1.6
Intermediate-Risk	887	8.0	12.2	1.1
Low-Risk	1724	7.4	11.5	2.2
Intermediate-Risk	155	9.2	12.0	1.6
High Risk	120	12.6	11.4	1.7
Low-Risk	321	5.5	15.1	2.2
Intermediate-Risk	400	7.7	11.5	1.3
Low-Risk	963	5.5	14.2	2.3
Low-Risk	214	5.2	12.3	1.2
Low-Risk	268	5.4	13.2	1.7
Low-Risk	635	6.3	13.0	2.1
Intermediate-Risk	87	8.0	10.1	1.3
Intermediate-Risk	108	5.6	12.5	0.6
High Risk	1707	7.5	12.1	1.0
High Risk	118	9.6	9.9	1.3
Low-Risk	1046	10.6	11.1	3.8
Low-Risk	353	6.2	12.0	1.5
High Risk	75	7.0	11.0	0.7
Intermediate-Risk	98	5.7	11.1	0.8
Low-Risk	304	11.6	11.5	3.5
Intermediate-Risk	153	8.9	12.7	1.4
Low-Risk	652	6.4	13.8	1.6
Intermediate-Risk	221	5.1	13.0	1.1
Intermediate-Risk	1152	7.4	15.1	1.2
Intermediate-Risk	872	11.3	16.0	2.1
Intermediate-Risk	273	3.2	11.2	0.6
High Risk	365	9.3	10.1	1.6
High Risk	1973	22.0	8.5	2.9
Intermediate-Risk	782	7.1	12.0	1.3
Intermediate-Risk	1968	6.8	11.5	1.6
Low-Risk	1758	3.8	9.8	1.0
High Risk	186	18.8	10.8	2.6
Intermediate-Risk	369	3.9	9.9	0.9
Low-Risk	387	4.3	11.9	1.0
Low-Risk	410	6.7	12.8	1.7
Low-Risk	195	4.5	13.4	1.3
Low-Risk	328	3.1	9.9	1.6
High Risk	785	16.0	11.6	2.4
Intermediate-Risk	233	7.4	11.8	1.0
Intermediate-Risk	189	9.0	7.0	1.8
Intermediate-Risk	621	4.0	12.8	0.9
Intermediate-Risk	200	6.5	12.0	1.2
Intermediate-Risk	96	8.0	13.0	1.7

Low-Risk	501	5.5	11.4	1.7
High Risk	185	7.5	11.4	1.4
Intermediate-Risk	491	8.1	12.1	1.7
Intermediate-Risk	363	6.0	12.5	1.3
Low-Risk	3289	8.9	14.0	2.2
Intermediate-Risk	706	4.5	10.1	0.9
Intermediate-Risk	242	6.9	12.3	1.5
Low-Risk	1333	8.6	12.5	2.7
Intermediate-Risk	1694	9.0	14.4	2.2
Low-Risk	546	5.1	11.9	1.6
Low-Risk	813	6.8	12.8	1.8
Low-Risk	2171	7.6	10.0	2.5
Low-Risk	123	7.0	13.6	1.7
High Risk	6	13.6	11.2	1.4
Intermediate-Risk	141	6.8	8.4	1.0
Intermediate-Risk	335	3.0	12.3	1.0
Low-Risk	117	4.6	11.8	1.4
Intermediate-Risk	774	4.7	10.1	0.5
Intermediate-Risk	1032	9.8	13.0	2.1
Low-Risk	353	5.7	12.3	1.6
Low-Risk	131	7.2	11.9	1.9
High Risk	31	16.3	11.5	1.7
Intermediate-Risk	140	8.1	13.1	2.7
Low-Risk	3912	2.7	12.8	1.6
Low-Risk	71	3.7	11.3	1.4
Low-Risk	514	3.1	9.8	0.8
Low-Risk	2550	8.2	10.7	2.0
Low-Risk	2926	6.5	14.0	1.8
High Risk	246	8.3	6.5	1.5
Low-Risk	455	5.8	10.4	1.6
Intermediate-Risk	570	9.5	9.4	2.1
Low-Risk	131	8.3	13.6	2.2
Low-Risk	417	6.3	9.6	1.6
Intermediate-Risk	392	8.4	10.7	1.9
Low-Risk	331	3.4	11.8	0.9
Intermediate-Risk	98	7.2	11.0	0.9
Intermediate-Risk	23	19.4	10.1	1.3
Low-Risk	1379	14.1	9.4	1.9
Low-Risk	414	9.1	12.9	3.0
High Risk	258	11.8	13.7	1.2
Intermediate-Risk	437	7.9	10.0	1.6
Intermediate-Risk	92	5.5	9.9	1.0
High Risk	463	23.3	9.9	1.4
Intermediate-Risk	1226	2.9	9.5	0.6
Intermediate-Risk	709	2.5	12.2	0.5
Low-Risk	317	7.5	12.9	2.2

Low-Risk	84	6.4	11.8	1.6
Intermediate-Risk	709	7.6	11.5	1.5
Low-Risk	746	4.5	10.1	1.4
Low-Risk	110	5.6	10.4	1.8
Low-Risk	493	1.7	12.3	0.8
Low-Risk	139	4.6	12.8	1.3
Low-Risk	156	3.0	10.8	2.5
High Risk	105	11.5	8.7	1.4
Low-Risk	2223	6.2	12.5	2.6
Intermediate-Risk	421	7.7	10.9	1.3
Intermediate-Risk	528	7.0	10.6	1.0
Intermediate-Risk	40	6.4	8.5	1.1
Low-Risk	1835	7.1	12.9	2.1
Low-Risk	516	8.2	12.1	1.9
Intermediate-Risk	597	10.0	11.0	1.3
Low-Risk	1316	10.1	14.4	2.6
Intermediate-Risk	335	5.0	9.7	1.1
Intermediate-Risk	1255	10.1	12.4	2.2
Intermediate-Risk	34	14.8	12.0	3.8
Intermediate-Risk	285	6.8	10.9	1.5
Intermediate-Risk	465	4.7	11.7	0.8
Intermediate-Risk	1527	10.6	10.4	2.3
Low-Risk	414	4.9	11.9	1.5
High Risk	604	11.2	10.3	1.5
Low-Risk	239	2.5	11.0	1.0
Intermediate-Risk	1118	3.0	8.9	0.9
High Risk	124	12.0	10.3	1.9
Low-Risk	82	8.0	13.1	2.1
Intermediate-Risk	39	2.4	8.0	0.6
Low-Risk	239	9.5	11.6	2.7
Low-Risk	253	4.8	9.4	1.4
Low-Risk	387	9.3	10.4	3.2
Intermediate-Risk	342	7.4	12.8	1.5
Low-Risk	385	4.0	10.9	1.1
Low-Risk	812	2.8	13.8	1.6
Intermediate-Risk	771	5.0	12.6	1.2
High Risk	1365	15.8	8.7	2.0
Intermediate-Risk	254	4.0	12.0	0.7
High Risk	44	12.1	8.7	1.7
Low-Risk	1377	4.2	12.7	1.1
Intermediate-Risk	215	34.4	9.8	3.4
Intermediate-Risk	22	4.8	11.4	0.7
Low-Risk	231	5.4	13.1	1.4
High Risk	251	8.3	10.8	1.2
Low-Risk	670	3.6	12.1	1.1
Intermediate-Risk	370	7.4	10.9	1.4
High Risk	115	15.2	10.3	1.7

High Risk	68	18.4	8.4	1.5
Low-Risk	103	6.0	11.3	1.6
Low-Risk	1495	6.7	9.7	1.9
Low-Risk	677	8.1	10.8	3.5
Low-Risk	400	6.9	12.0	2.3
High Risk	206	15.0	11.1	1.7
High Risk	479	24.5	9.3	2.1
Low-Risk	726	4.5	11.5	1.5
Intermediate-Risk	489	4.6	9.9	0.8
Intermediate-Risk	547	5.7	13.2	1.0
Intermediate-Risk	767	7.2	14.3	1.6
Intermediate-Risk	1205	5.5	10.3	1.2
Intermediate-Risk	131	11.5	10.3	1.9
Low-Risk	2725	2.1	11.2	1.2
Low-Risk	326	11.8	10.1	3.1
Intermediate-Risk	153	8.2	11.9	1.7
Intermediate-Risk	350	9.8	11.3	1.9
Low-Risk	1164	5.0	13.4	1.3
Low-Risk	1248	7.5	13.2	3.2
Low-Risk	434	3.2	8.2	1.1
Intermediate-Risk	311	7.6	11.3	1.2
Low-Risk	898	5.5	11.5	1.7
High Risk	414	9.5	13.6	1.6
Intermediate-Risk	276	7.9	12.7	1.6
Low-Risk	43	7.7	10.9	1.8
High Risk	54	9.3	10.0	1.2
Intermediate-Risk	2522	10.1	15.7	1.6
High Risk	36	16.9	11.4	1.5
Intermediate-Risk	75	5.6	9.0	1.2
Low-Risk	608	7.5	14.0	1.9
Intermediate-Risk	368	6.1	14.2	1.2
Intermediate-Risk	136	8.2	14.2	1.6
High Risk	75	13.7	13.2	1.4
Low-Risk	409	11.4	14.0	2.8
Intermediate-Risk	33	15.0	10.4	3.7
Low-Risk	188	4.3	12.2	1.6
Intermediate-Risk	306	6.3	13.5	1.1
Intermediate-Risk	327	9.8	14.3	2.2
Intermediate-Risk	766	9.0	15.2	1.4
Low-Risk	516	7.4	14.2	2.8
High Risk	14	15.0	10.8	0.7
Intermediate-Risk	211	11.6	12.9	1.6
High Risk	107	12.6	14.4	1.7
Intermediate-Risk	583	6.4	11.7	1.3
Low-Risk	1045	11.4	14.3	2.8
Intermediate-Risk	1939	7.6	12.8	1.1

High Risk	19	11.2	11.2	1.6
Low-Risk	416	8.6	14.4	2.5
High Risk	57	15.3	15.0	1.4
Low-Risk	1142	4.3	10.9	1.3
Low-Risk	566	4.4	11.1	1.2
Low-Risk	392	8.2	12.9	2.0
Low-Risk	796	6.5	11.5	1.9
Intermediate-Risk	682	8.3	12.7	1.4
Low-Risk	385	5.4	13.3	1.6
Intermediate-Risk	525	8.3	14.6	2.2
High Risk	46	10.9	11.1	1.8
Intermediate-Risk	50	10.8	13.7	2.0
Low-Risk	240	5.6	14.2	1.3
High Risk	117	15.4	11.4	1.3
High Risk	21	10.0	9.9	0.9
Intermediate-Risk	334	7.7	14.7	1.2
Low-Risk	422	6.9	11.4	1.9
Intermediate-Risk	91	7.2	10.6	1.4
Intermediate-Risk	2240	4.1	9.6	0.8
Low-Risk	94	7.4	15.1	2.8
High Risk	43	14.2	10.4	3.1
Low-Risk	59	5.6	11.7	2.0
Intermediate-Risk	499	9.1	12.3	1.8
Low-Risk	2137	3.9	11.3	1.2
Intermediate-Risk	710	6.6	12.9	1.4
Intermediate-Risk	291	10.2	15.9	1.9
Intermediate-Risk	645	9.5	13.9	1.0
Low-Risk	565	6.4	11.6	1.8
Low-Risk	219	6.3	10.8	1.7
Intermediate-Risk	166	7.6	11.1	1.4
High Risk	43	9.5	9.6	0.7
High Risk	62	10.4	11.6	2.0
Intermediate-Risk	189	16.9	10.9	1.6
Intermediate-Risk	129	7.1	7.9	1.2
Intermediate-Risk	174	8.8	9.5	2.2
Intermediate-Risk	201	7.0	13.8	1.2
Low-Risk	106	5.1	14.7	2.3
Intermediate-Risk	1335	4.4	11.4	1.0
Intermediate-Risk	14	2.9	9.7	0.9
Low-Risk	1043	8.7	10.0	2.3
Intermediate-Risk	352	73.3	12.3	1.5
Low-Risk	2303	8.1	11.5	2.7
Low-Risk	251	3.3	10.9	1.1
Intermediate-Risk	86	6.9	10.2	0.9
High Risk	28	12.5	8.7	0.9
Low-Risk	555	9.5	14.6	3.6
Intermediate-Risk	193	7.4	12.7	1.1

Low-Risk	164	6.0	9.4	1.7
Intermediate-Risk	280	7.9	11.2	1.4
Intermediate-Risk	151	6.4	9.8	0.8
Low-Risk	1983	3.3	12.0	0.9
Intermediate-Risk	436	8.6	12.3	1.9
Low-Risk	3289	4.8	11.7	1.6
High Risk	19	8.2	9.2	1.0
High Risk	85	8.3	12.2	0.6
Intermediate-Risk	287	7.1	11.1	0.9
Low-Risk	813	4.5	9.4	2.0
Low-Risk	390	4.6	10.5	1.9
Intermediate-Risk	171	7.1	7.9	0.4
Intermediate-Risk	280	5.9	10.3	0.8
Intermediate-Risk	413	6.3	11.3	1.2
Intermediate-Risk	15	8.3	11.6	0.6
Intermediate-Risk	147	4.3	8.6	0.4
Low-Risk	488	4.4	12.5	1.4
Intermediate-Risk	40	9.2	6.7	2.2
Intermediate-Risk	372	6.1	11.8	1.1
Intermediate-Risk	379	7.6	13.4	1.6
High Risk	14	10.9	8.3	1.3
Intermediate-Risk	479	9.3	10.9	2.0
Low-Risk	279	6.0	13.5	1.9
High Risk	29	15.4	14.2	1.3
High Risk	23	23.9	9.7	1.8
Low-Risk	1	6.9	13.1	2.0
Low-Risk	366	9.9	12.9	2.5
High Risk	34	7.3	8.9	1.0
High Risk	688	26.5	8.9	3.3
Intermediate-Risk	151	9.2	9.0	1.8
Low-Risk	172	3.9	9.9	1.0
Intermediate-Risk	1074	6.5	13.0	0.6
Intermediate-Risk	103	5.1	9.1	1.2
