# Supplementary material for: Actionable tests and treatments for patients with gastrointestinal cancers and historically short median survival times
Source: PLoS One. 2022 Nov 2;17(11):e0276492. doi: 10.1371/journal.pone.0276492 (PMC9629612; doi:10.1371/journal.pone.0276492)
Supplement: Attachment — Submitted filename: Response to Reviewers.docx [file pone.0276492.s002.docx]

**PLOS Rebuttal Letter**

**Response to the Reviewers:**

Thank you for the reviewers’ efforts. They are sincerely appreciated. The authors agree with the recommendations. Our responses below and text modifications address each requirement and question.

The modifications and clarifications contribute to the manuscript and support our message and conclusions. Please see our revised copy with the track changes.

**Response to Journal Requirements:**

1. Our manuscript now meets PLOS ONE’s style requirements including those for file naming.
2. We removed funding information from the acknowledgements section of our manuscript and approve of it being written as follows:

Funding: The Marcus Foundation, MZB Foundation for Cancer Research, and Aid L’Shalom Foundation. The funders had no role in study design, data collection and analysis, decision to publish, or preparation of the manuscript, with the exception of Dr. Howard Bruckner as the Chief Science Officer of the MZB Foundation.

3. The newly created ORCID for the corresponding author is up to date and entered in the Editorial Manager. Please see the attached link for your reference: Howard W Bruckner, HW Bruckner (0000-0002-7459-7255) (orcid.org)
4. A full ethics statement appears in our Methods Section and describes the consent as written. It was designed and approved by the IRBs.

**Reviewer #1 – Comments to Reviewer:**

1. The title of the paper clarifies the term by replacing “difficult” with “historically short median survival times.”
2. The abstract now reflects the study and its results. Line 44 in the previous manuscript was removed in the revised copy due to space limitations.
3. The introduction is consolidated. Parts of the introduction that must be in the methods section were moved there to avoid duplication. The introduction now starts with the background of historical gastrointestinal survivals and adverse event experiences. Added information provides rates of American and worldwide patients that die per annum. These are the patients that have these unmet needs each year. The function and objectives of the A.L.A.N. Score are described in the introduction, in lines 114-125. The methods of the A.L.A.N. Score are now described in the methods section, in lines 165-169.
4. This is a clinical trial conducted in an outpatient oncology clinic. The patients were treated in a single practice, coordinated with hospital IRBs. The trial was expanded with IRB approvals because of the compassionate unmet needs and additional objective of more information, identification of the characteristics of patients that will gain benefit, and therapeutic hypotheses for investigation. This information was placed in the Methods section, in lines 212-214.

The selection process occurred over a 2-year period, as stated in line 211. The cut off dates avoid the need to parse the impact of interruptions in referral and treatment due to the pandemic.

Intent-to-treat analyses (select) evaluate the most difficult patients in order to demonstrate proof of principal, with the most difficult, stage-4 diagnoses, and patients with unmet needs. These are the series for which we have adequate numbers, 36-50 patients in each group, not anecdotes. The information regarding the prognostic blood tests is new and applicable to unmet needs for patients with resistant colorectal cancer and cholangiocarcinoma. Physicians need to know and be able to identify patients that can often benefit from treatment.

Ineligibility is minimized because the objective is to provide treatment to patients with unmet needs. Our regimen has twenty years of safety due to low dosage. Exclusion ineligibility criteria prioritize safety, office care, and criteria that are potentially suitable for institutional cancer centers and cooperative groups. This was a deliberate effort to minimize exclusion in order to test and cautiously expand limits of eligibility. Prior experience with this project evolved in step wise fashion due to an ever-increasing volume of referrals for “untreatable, last resort” patients. We were pressured to continue to test the limits of safety and eligibility because safety allows treatment and the benefit rate of > 50% encouraged continuation.

The reason to omit standard practice is because independent expert consultants advised against it because they anticipated median survival times of < 10 months due to diagnoses stage, volume of disease, and complications. This was added into the manuscript and explained in the methods section, lines 176-178.

The practice adheres to NCI grade blood tests as used by the cooperative groups because they make sense and avoid a level of complexity that would preclude adoption by other investigators. This was explained in the Methods section, in lines 171-172. The grade 0-2 blood tests are defined in reference 30.

5. The different series are clearly explained. Line 287 in the previous manuscript which is Line 278 in the revised copy now reads the correct numbers, including an HR of 2.96 and P value of 1.2x10^-6^.
6. Discussion: individual tests that predict favorable survival are shown in Table 3. These include: high serum albumin, high NLR, low ANC, low platelets, low alkaline phosphates, high lymphocytes, high hemoglobin, and high LMR. The favorable test groups for resistant advanced pancreatic cancer patients are enumerated in the text. The discussion briefly mentioned it because they demonstrate the consistent potential utility of the prognostic blood tests (This is applying to more heavily treated groups, with applications to ~ 80% of patients that are striking novel demonstrations.) The tests that predict short MST are now included in line 374.

- Line 320 in the previous manuscript, now line 366 in the revised copy: The 73-83% of patients with an AS of 0-2. This was simplified to 81%.
- Line 321 in the previous manuscript, now line 367 in the revised copy: The 41.6 patients. This was simplified to the 42% of the patients with A.L.A.N. Scores of 0 – 2.
- Line 322 in the previous manuscript, now line 368 in the revised copy: MSTs of ≥ 16 to ≥ 24 months. This was simplified to say ≥ 16 months.
- Table 1: 2-year survival rate and CI for all the patients were corrected.

**Reviewer #2 – Comments to Reviewer:**

- Line 28 in the previous manuscript: Due to space limitations and to avoid repetition, this line was integrated into the conclusion in lines 48-49.
- Line 110 in the previous manuscript: The A.L.A.N. Score consists of the number, 0-4, of individual unfavorable tests. This was clarified by having the paper read “and also individual tests” in line 114. The correct reference was also placed in the revised manuscript. We encorporated the reviewer’s A.L.A.N. score clarification into our introduction, in lines 114-125. We explain the model, how it is scored, the disease Cholangiocarcinoma, and the illustrative result.
- NLR has the value of 3.0, now corrected in the manuscript. The reference at the end of the sentence, line 118, is now corrected (reference 29).
- Line 116 in the previous manuscript, line 121-122 in the revised copy: RAPC’s preferred abbreviation is inserted.
- Table 1: The word “years” is added to clarify that < 70 and ≥ 70 refers to age.
- The spelling of cholangiocarcinoma was corrected in the Figure 1 legend.
- In the results section, lines 297-300, we describe a sensitivity analysis omitting cholangiocarcinoma. We also included a line explaining that the A.L.A.N. score and individual tests produced similar findings for each disease.
- The “observed” disparities between resistant and non-resistant A.L.A.N. Score 3-4 patients may be due to several factors. First, the small number of patients are not significant. Second, there may hypothetically be a selection with inadvertent referral differences. Patients with no prior treatment may have a lower threshold, worse AS 3-4, whereas A.L.A.N. Score 3-4 patients with prior treatment are less likely to be referred for another treatment. This is illustrated when the groups are matched for the most powerful individual tests; the difference disappears, but the numbers remain exploratory. This was not discussed in the manuscript unless it is the reviewers’ recommendation to do so.
- **Ultimately the paper is examining primarily the predictive value of the A.L.A.N. score in patients receiving GFLIO chemotherapy. It would seem to be a more straightforward title and thrust to the paper.** We agree with this statement; however, we want to make the point of expanding the tests to patients with unmet needs due to short survival, treatment resistance, and age.
